# The Relationship Between Vitamin E Plasma and BAL Concentrations, SOD Activity and Ventilatory Support Measures in Critically Ill Patients

**Published:** 2011

**Authors:** Shadi Ziaie, Hamidreza Jamaati, Mannan Hajimahmoodi, Seyyed Mohammadreza Hashemian, Fanak Fahimi, Behrooz Farzanegan, Ghazaleh Moghaddam, Golnar Radmand, Behzad Vahdani, Seyed Alireza Nadji, Sarah Mousavi, Hadi Hamishehkar, Mojtaba Mojtahedzadeh

**Affiliations:** a *Department of Clinical Pharmacy, Faculty of Pharmacy, Tehran University of Medical Sciences, Tehran, Iran.*; b *Tobacco Prevention and Control Research Center, NRITLD, Masih Daneshvari Hospital, Shahid Beheshti University of Medical Science, Tehran, Iran.*; c *Department of Drug and Food Control, Faculty of Pharmacy, Tehran University of Medical Sciences, Tehran, Iran.*; d *Nursing and Respiratory healt Managment Research Center, NRITLD, Masih Daneshvari Hospital, Shahid Beheshti University of Medical Science, Tehran, Iran.*; e *Chronic Respiratory Disease Research Center, NRITLD, Masih Daneshvari Hospital, Shahid Beheshti University of Medical Sciences, Tehran, Iran.*; f *Department of Clinical Pharmacy, Faculty of Pharmacy, Shahid Beheshti University of Medical Sciences, Tehran, Iran.*; g *National Research Institute of Tuberculosis and Lung Disease (NRITLD), Masih Daneshvari Hospital, Shahid Beheshti University of Medical Sciences, Tehran, Iran. cDepartment of Drug and Food Control, Faculty of Pharmacy, Tehran University of Medical Sciences, Tehran, Iran.*; h *Virology Research Center, NRITLD, Masih Daneshvari Hospital, Shahid Beheshti University of Medical Sciences, Tehran, Iran.*; i *Department of Clinical Pharmacy, Faculty of Pharmacy, Tabriz University of Medical Sciences, Tabriz, Iran.*

**Keywords:** Vitamin E, SOD, Antioxidant, Oxidative stress, Mechanical ventilation

## Abstract

Vitamin E is a potent reactive oxygen metabolites (ROM) scavenger. It is a lipid-soluble vitamin and its main function is to protect polyunsaturated fatty acids against oxidative stress.

Twenty-five mechanically ventilated Intensive Care Unit (ICU) adult patients participated in a prospective randomized clinical trial receiving either placebo (10 patients) or 3 IM doses (1000 IU each) of vitamin E (15 patients). We determined plasma and bronchoalveolar lavage (BAL) fluid concentrations of vitamin E and superoxide dismutase (SOD).

Among these 25 patients, there were 14 men and 11 women, aged 63.16 ±15.48 years (mean ± SD; range = 33 to 87 years). Vitamin E supplementation resulted in significant differences in plasma and BAL vitamin E concentrations between the two groups (p-value = 0.01, 0.01), decrease in SOD activities (not differ significantly in plasma (p-value = 0.23)), but with significant differences in BAL (p-value = 0.016) and progressive reduction in Acute Physiology and Chronic Health Evaluation II (APACHE II) (p-value = 0.52) and Sequential Organ Failure Assessment (SOFA) (p-value = 0.008) score in vitamin E group.

From the results of this study, it seems that supplementation of vitamin E as a potent antioxidant, along with other supportive measures, can be beneficial in decreasing SOD total activity, ROM production and risk of organ failure in critically ill patients.

## Introduction

Oxidative stress happens in critical illnesses, specifically in sepsis, shock, Acute Respiratory Distress Syndrome (ARDS) and Disseminated Intra-vascular Coagulation (DIC), due to the reactive oxygen species (ROS) and reactive nitrogen species (RNS) (1-5). Oxygen is necessary for the metabolic production of energy in the form of ATP. Under normal conditions, during this process, O_2_ is reduced to H_2_O and produce several intermediate ROS (5) but in critically-ill patients, ROS production will be increased, triggering the release of cytokines, activating inflammatory cascades and increasing the expression of adhesion molecules and ischemia/reperfusion-induced tissue damage (1, 6, 7). Therefore, the administration of high O_2_ concentrations would be detrimental to mechanically ventilated ICU patients (5). Level of plasma and intra-cellular antioxidants and free electron scavengers, also the enzymes activity involved in ROS detoxification, are decreased in critically ill patients such as sepsis and ARDS (5, 8, 9) and these oxidant/antioxidant imbalances have a main contribution in the pathogenesis of oxidative stress and multiple organ failure (10-14). In ARDS, characterized through non-cardiogenic pulmonary edema, cytokines release and neutrophils influx, which requires the use of mechanical ventilation with high levels of oxygen for adequate brain and other vital organs oxygenation, prolonged exposure to hyperoxia can damage pulmonary epithelial cells (15).

Circulatory antioxidant deficiency and increase in oxidative stress and MOFs have been associated with greater morbidity and mortality in mechanically-ventilated patients (16, 17).

Recent clinical studies reported that prophylactic administration of antioxidant supplements, as a component of nutritional support or as an individualized intervention, with ideal concentrations at the right place and the right time, (14, 18) can be effective in terminating the activity of ROS, lowering oxidative stress and its related complications (5, 17, 19-21).

Vitamin E is a potent reactive oxygen metabolites (ROM) scavenger especially in the lung (18, 22). It is a lipid-soluble vitamin and its main function is to protect polyunsaturated fatty acids (PUFA) against oxidative stress. It can break the chain, prevent lipid peroxidation, trap peroxyl free radicals and prevent disruption of the membrane integrity (19, 23). The effect of enteral vitamin E supplementation has been studied in various investigations (18, 24-26).

Antioxidant enzymes, including superoxide dismutase, catalase and glutathione peroxidase, are a group of enzymes that reduce reactive oxygen metabolites. SOD converts superoxide anion (O^.^_2_^-^) to O_2_ and H_2_O_2_; catalase and glutathione peroxidase degrade H_2_O_2_ to H_2_O. When the oxidative stress process is active, the amount of ROM will increase and result in cellular damage. The antioxidant enzymes and non-enzymes are linked to one another (21), therefore, the activity of these antioxidant enzymes will also be increased, respectively (19). Recent trials showed that pretreatment with SOD protected against oxygen poisoning in patients receiving oxygen in high concentration (5, 27). Due to the short half-lives of ROS, clinically their measurement is difficult. Therefore, vitamin E − as a secondary reactive product-can be used to quantify the oxidative stress (24).

The aim of this study was to compare the effects of antioxidant administration of vitamin E and placebo on SOD activity in plasma and bronchoalveolar lavage (BAL) fluid in mechanically-ventilated ICU patients.

## Experimental

*Methods*

In a prospective randomized clinical trial, in Masih Daneshvari Teaching Hospital (Shahid Beheshti University of Medical Sciences), 28 intensive care unit (ICU) adult patients with at least 72 h on mechanical ventilation, having fraction of inspired oxygen (FiO_2_) > 50% and positive end expiratory pressure (PEEP) > 5 cm H_2_O, were entered into our study. Three patients were eliminated because of death during the first day of study. After randomization into two groups, 15 patients received vitamin E (vitamin E group) and 10 received placebo (placebo group).

Ritten informed consent was obtained from each patient before inclusion. The study protocol was approved by the Institutional Ethics Committee. 

Patients with a history of warfarin use, vitamin E substitution, or any other antioxidant therapy (selenium, vitamin C and A) from the first day of ICU admission, impaired renal function (creatinine > 2.5 mg/dL, doubling of creatinine, urine output < 0.5 cc/Kg/h or dialysis), blood pressure < 90/60 mmHg ( Mean Arterial Pressure (MAP) < 65 mmHg ), blood pH < 7.2, platelet count below 50000 or active bleeding preceding entrance to the study, were excluded. Vitamin E was given as IM injection of 1000 IU each day, for 3 days. Placebo was the same volume (10 cc) of normal saline.

Blood samples were taken at the following timepoints: baseline and 3 h and 24 h after each injection. Great caution was exercised to prevent hemolysis. The plasma of each sample was obtained through centrifugation at 10°C, and stored at –80°C before determination of vitamin E, SOD activity and contents. Bronchoalveolar lavage fluid was taken at baseline and 24 h after the last injection. Each sample was centrifuged at 10°C and stored at –80°C for determination of vitamin E, SOD activity and contents. Blood samples were taken at baseline and also daily till the end of the study (7 days) for determination of full chemical and metabolic parameters, including Blood Urea Nitrogen (BUN), creatinine, Complete Blood Count (CBC) and platelets, Arterial Blood Gas (ABG), sodium, potassium and bilirubin. Clinical parameters including blood pressure, temperature, heart rate, respiratory rate and Glasgow Coma Scale (GCS) were also determined.

*Determination of Vitamin E*

Concentrations of vitamin E in plasma and BAL fluid were determined through the high-performance liquid chromatography (HPLC) equipped with UV detector at 294 nm. One hundred and fifty μL plasma samples were deproteinized with retinol acetate in ethanol and extracted in *n*- hexane. After evaporation to dryness under nitrogen, the residue was dissolved completely in methanol : butanol (95 : 5). Methanol : butanol (95 : 5) was used as mobile phase and the column used was Eurospher 100 C8 (4.6 mm × 25 cm). The flow rate was set at 1.0 mL/min and retinol acetate was used as internal standard.

*Determination of SOD activity*

Plasma and BAL fluid SOD activity was determined through SOD assay kit (Cayman Chemical Company, USA). In this colorimetric based assay, superoxide ions were generated from the conversion of xanthine and oxygen to uric acid and hydrogen peroxide through xanthine oxidase. The superoxide anion then converted tetrazolium salt to formazan, a colored product that absorbs light at 450 nm. SOD reduces the superoxide ion concentration and thereby, lowers the rate of formazan formation. Reduction in the appearance of formazan dye is a measure of SOD activity. In other words, relative SOD activity is determined from percent inhibition of the rate of formation of formazan dye.

The following clinical outcomes were recorded:

Acute Physiology and Chronic Health Evaluation II (APACHE II)

Sequential Organ Failure Assessment (SOFA) score for 7 days

*Statistics*

Statistical analysis was done through repeated-measures analysis of variance (ANOVA), using SPSS 16. When the requirements for a parametric group comparison were not met, the corresponding non-parametric test was conducted. For patient characteristics, the chi-square and the Mann-Whitney tests were used for categorical and continuous data, respectively. The t-test for paired data was used for comparison between two groups. Data are expressed as mean ± SD. A p-value < 0.05 was considered significant.

## Results and Discussion

Twenty-five patients, 15 in the vitamin E group and 10 in the placebo group, completed the study. Among these, were 14 men and 11 women, aged 63.16 ± 15.48 years (mean ± SD; range = 33 to 87 years).

Both groups received standard supportive treatments for critically-ill patients. The treatments were well tolerated and no adverse events were recorded.

The co-morbid conditions were observed including ARDS (n = 3; 12%), Cerebral Vascular Accident (CVA) (n = 1; 4%), Chronic Obstructive Pulmonary Disease (COPD) (n = 9; 36%), Loss Of consciousness **(**LOC) (n = 1; 4%), corpulmonale (n = 1; 4%), lung cancer (n = 4; 16%), Amyotrophic Lateral Sclerosis **(**ALS) (n = 1; 4%), sepsis (n = 2; 8%), sagittal sinus thrombosis (n = 1; 4%), post Mitral Valve Replacement (MVR) (n = 1; 4%) and tracheal stenosis (n = 1; 4%).

Demographic and clinical data of all patients are shown in Table 1.

**Table 1:** Base line patient characteristics comparing vitamin E with placebo groups

	**Vitamin E group**	**Placebo group**	**p- value**
**Patients **	15	10	
**Age, yr**	64.53 ± 17.48	61.10 ± 12.47	0.59
**Male/Female**	9/6	5/5	0.69
**APACHE II Score**	24.20 ± 4.80	20.60 ± 4.40	0.06
**SOFA Score**	8.60 ± 1.90	7.20 ± 2.00	0.12
**Plasma Vit E, mcg/mL**	9.90 ± 1.40	9.30 ± 1.30	0.31
**BALF Vit E, mcg/mL**	2.00 ± 0.50	1.70 ± 0.60	0.10
**Plasma SOD, mcg/mL**	0.32 ± 0.14	0.38 ± 0.11	0.27
**BALF SOD, mcg/mL**	0.19 ± 0.20	0.14 ± 0.13	0.41

Data are expressed as mean ± SD. APACHE: Acute physiology and chronic health evaluation; SOFA: Sequential organ failure assessment; BALF: Bronchoalveolar lavage fluid; SOD: Super oxide dismutase

The vitamin E concentrations in plasma and bronchoalveolar lavage were not different at baseline in the two groups (p- value = 0.31, 0.10); moreover, there was no significant alteration from the first to seventh measurement in each group (p- value = 0.30 in vitamin E group and 0.46 in placebo group).

**Figure 1:**
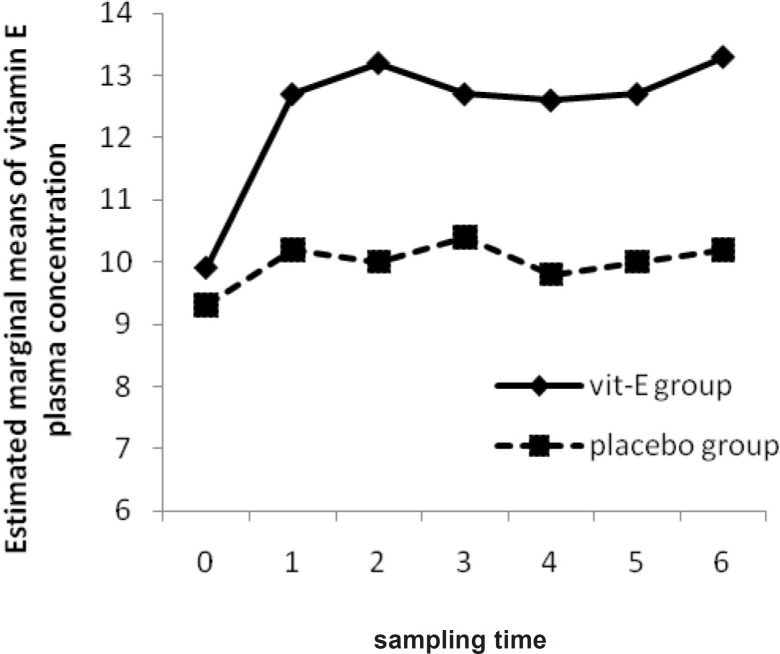
The Mean of Vitamin E plasma concentration in vitamin E and placebo groups versus sampling times

However, there was a significant difference in plasma and BAL vitamin E concentrations between vitamin E and placebo group after the last measurement (p-value = 0.01, 0.01) (Figure 1). Plasma SOD activities of vitamin E group were not significantly altered during the first to seventh measurement (p-value = 0.27). Furthermore, there was no significant difference in plasma SOD activities of placebo group during the study (p-value = 0.83). Between the two groups, plasma SOD activities did not differ significantly (p-value = 0.23) unlike the BAL SOD activities (p-value = 0.01).

**Figure 2:**
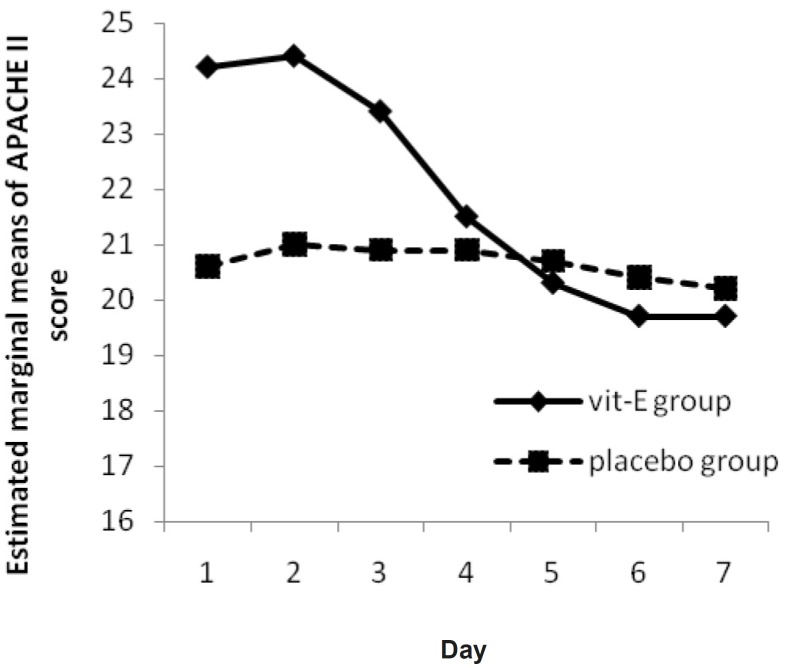
The Mean of APACHE II Score in vitamin E and placebo groups versus day

No significant changes were observed in APACHE II score over time, neither in each group (p-value = 0.52 in vitamin E group and 0.46 in placebo group), nor between them (p-value = 0.20) but a progressive reduction in APACHE II score was observed in vitamin E group (Figure 2).

**Figure 3:**
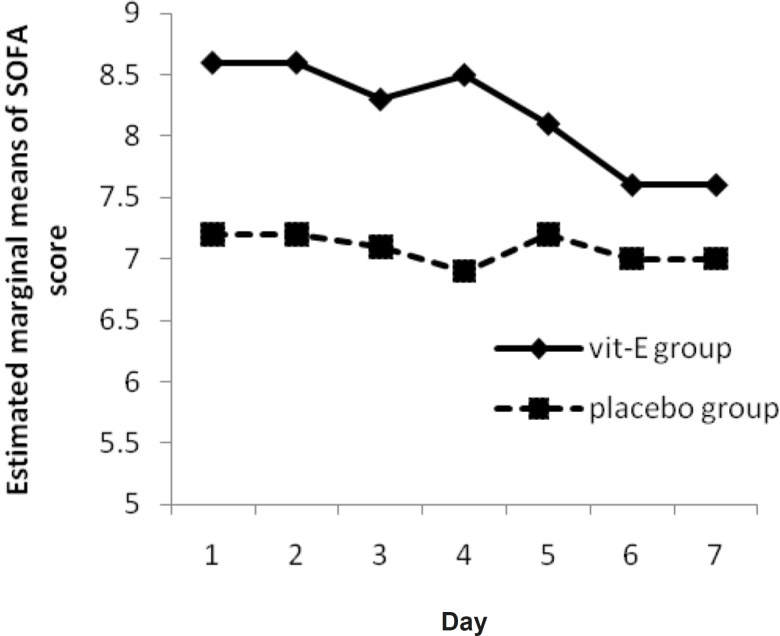
The Mean of SOFA Score in vitamin E and placebo groups versus sampling times

A significant reduction was observed in SOFA score in vitamin E group (p-value = 0.008) (Figure 3). There was also a significant difference between the two groups (p-value = 0.04).

In mechanically ventilated ICU patients, due to the toxic effect of high O_2_ concentration (5), prolonged mechanical ventilation and exposure to hyperoxia can damage pulmonary epithelial cells (15). In these patients, level of plasma and intra-cellular antioxidants and free electron scavengers, as well as the enzymes activity involved in ROS detoxification, is decreased (5, 8, 9) and these oxidant/antioxidant imbalances have a main contribution in the pathogenesis of oxidative stress and MOFs (10-14). 

This study was designed to determine the antioxidant effect of vitamin E supplementation on SOD activity in mechanically-ventilated ICU patients. 

Enteral feeding is the common method of vitamin E administration, but in acute clinical situations, such as in ICU patients, oral administration of vitamin E is not effective as drug absorption may be impaired in this patient population (20) and a long period, almost 14 days, is needed for a sufficient increase in endogenous vitamin E concentration (22). As a result, for high-risk patients, administration of parenteral formulations, like IM administration, would be an advantage.

Owing to short half-lives of ROS, clinically their measurement is difficult (23). On the other hand, vitamin E is an integral constituent of the lung surfactant and acts as an antioxidant in the lung (22). Therefore, vitamin E concentration in plasma and bronchoalveolar lavage fluid, as a secondary reactive product, can be used to quantify oxidative stress (23). Measurement of antioxidant defenses, in one study, has demonstrated depressed plasma levels of vitamins E and C in patients with sepsis and ARDS (21) but in our study, like another study on severe COPD patients (28), mean plasma vitamin E concentration in all patients was 9.70 ± 1.30 mcg/mL at base line which is within the normal range compared to vitamin E concentration in normal subjects (29). Vitamin E, in addition to its activities as an antioxidant, is involved in immune function, therefore, it is expected that immune defense be increased with exogenous vitamin E supplementation (29). Besides, in ARDS patients, oxidative stress markers are elevated several folds in plasma and BALF (30-32), so, much higher physiological requirement of vitamin E is needed.

In all patients, Mean BAL fluid vitamin E concentration was 1.90 ± 0.50 mcg/mL at base line. Analysis of antioxidant compounds may be difficult through the overall low levels of substances in BALF, due to the 100-200 fold dilution of epithelial lining fluid (ELF) during the lavage procedure (32). In this study, vitamin E was given as IM injection of 1000 IU each day, for 3 days. The increase in vitamin E concentration in plasma and BAL fluid resulted in significant differences between two groups (Figure 1). Whether a longer duration of vitamin E supplementation would provide, some benefit needs to be investigated. However, the augmentation of antioxidants noted through vitamin E in earlier reports, provides a rationale for further exploration of its therapeutic potential (28) during the high concentration of oxygen therapy (FiO_2_ ≥ 50%). Although this vitamin is very safe and the high concentrations of oral intake even up to 3200 IU per day is tolerable without any side effects (29), dosing adjustment of vitamin E must be considered due to an increase in intra-thoracic pressure and a decrease in cardiac output accompanied by the use of mechanical ventilation (18).

In this study, the hypothesis that vitamin E administration would influence the APACHE II Score was analyzed through repeated measure ANOVA during 7 days. Although the APACHE II Score trend for vitamin E group shows a progressive reduction (Figure 2), it was not significant neither in each group nor between them. In other words, routine treatment protocol improved the situation in both groups by time. Treatment group recovery (as seen as its slope) was better, but differences were not significant (p-value = 0.20).

In a study by Hajimahmoodi *et al*., significant changes were observed in APACHE II score from first to seventh measurement (p = 0.0001) in treatment group (18). This difference might be due to the difference in APACHE II score determination time and different base line APACHE II score between the two groups. Another hypothesis that vitamin E supplementation would influence the SOFA score, was analyzed by Friedman’s non-parametric repeated measures during 7 days. Results for vitamin E group shows a significant progressive reduction (p-value = 0.008) (Figure 3) and there is a significant change between vitamin E and placebo group, in clinical severity of illness (as indicated by SOFA score) over the study period (p-value = 0.04).

Abiles *et al. *showed that oxidative stress markers do not worsen in patients with antioxidant vitamin intake even among those with greater deterioration in SOFA score (1).

**Figure 4:**
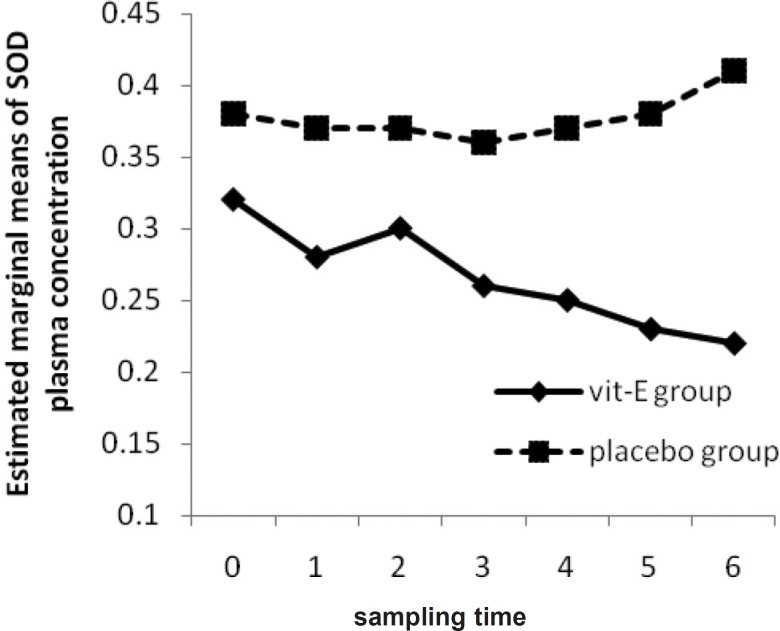
The Mean of SOD plasma concentration in vitamin E and placebo groups versus sampling times

Due to the activation of SOD activity being a characteristic feature of ROM production (19), we hypothesized that the use of vitamin E as a potent ROM scavenger, along with other supportive treatments, is beneficial in decreasing SOD total activity. Analyzing the between-subject effects (placebo to vitamin E group) showed a p-value of 0.23 in plasma and a p- value of 0.01 in BAL. Through Friedman’s non-parametric assessment, with progressive reduction in plasma SOD activity in vitamin E group (Figure 4), the reduction of activity was not significant neither in vitamin E (p-value = 0.27) nor in placebo group (p-value = 0.83). However, plasma SOD activity is significantly different at 3 and 24 h after the 2^nd^ and 3^rd^ vitamin E administration between vitamin E and placebo group (p-value = 0.04, 0.04, 0.007, 0.001).

Akiyama *et al. *showed that there was no significant difference in SOD activities between two groups during the study but dialysis under vitamin E-coated membrane or oral vitamin E supplementation, could significantly reduce Cu/Zn-SOD content in plasma and due to direct SOD protective effect against ROM, vitamin E appeared to be an appropriate agent to decrease hemodialysis-induced oxidative stress (19). It has been showed in another study that scavengers, like vitamin E, play a very important role in terminating the activity of ROM through augmentation of antioxidant defenses (5) and can result in decreasing plasma and BAL SOD activity.

In our study, due to the limited sample size, little differences between groups would not be detected as significant.

The current results suggest that the severity of the illness process in ICU patients (as indicated by SOFA score) might be decreased with vitamin E supplementation, but further study with a larger sample size is necessary to investigate the vitamin E effect on SOD activity and content and its action in mechanically-ventilated patients under the conditions of oxidative stress.
